# PdRuO_2_/PVP nanomaterial as a highly selective, stable, and applicable potentiometric sensor for the detection of Cr^3+^

**DOI:** 10.1007/s00604-024-06543-6

**Published:** 2024-07-18

**Authors:** Kenan Çevik, İlyas Yildiz, Adnan Yildiz, Mehmet Salih Nas, Mehmet Hakki Alma, Mehmet Harbi Calimli

**Affiliations:** 1 Department of Secondary Science and Mathematics Education, Department of Chemistry Education, Faculty of Education, Yuzuncu Yil University, Van, Türkiye; 2 Department of Molecular Biology and Genetics, Tokat Gaziosmanpasa University, Tokat, 60000 Türkiye; 3 Research Laboratory Application and Research Center (ALUM), Iğdır University, Igdir, TR 76000 Türkiye; 4 Department of Organic Agriculture Management, Faculty of Applied Sciences, Igdir University, Igdir, TR 76000 Türkiye; 5 Department of Medical Services and Techniques, Tuzluca Vocational School, Iğdır University, Igdir, TR 76000 Türkiye

**Keywords:** Potentiometry, Sensor, Ionophore, Chromium(III), Nanomaterial

## Abstract

PdRuO_2_/PVP nanomaterial was synthesized using a straightforward method and characterized using advanced analytical methods such as TEM, XRD, XPS, elemental mapping and SEM. The synthesized PdRuO_2_/PVP nanomaterial was used as an ionophore in potentiometric sensor electrodes and successfully adapted to Cr^3+^ ion detection in a large number of aqueous samples. Several experimental parameters of the PdRuO_2_/PVP sensor such as potentiometric behavior, selectivity, repeatability, response time, pH, titration, and recovery in real samples were investigated. Potentiometric behavioral characteristics were performed in the concentration range 1 × 10^−6^–1.0 × 10^−1^ M. The repeated experiments performed six times showed that there was no deviation in the measurements. The limit of detection of the PdRuO_2_/PVP potentiometric sensor was very low with a value of 8.6 × 10^−8^ M. The potentiometric measurements showed that the synthesized PdRuO_2_/PVP ionophore was highly effective in detecting Cr^3+^ in a wide pH range of 2.0–8.0 and was found to have a shelf life of over 1 year. As a result, the synthesized PdRuO_2_/PVP electrode material was found to be highly selective, stable, and applicable for Cr^3+^ detection.

**Graphical Abstract:**

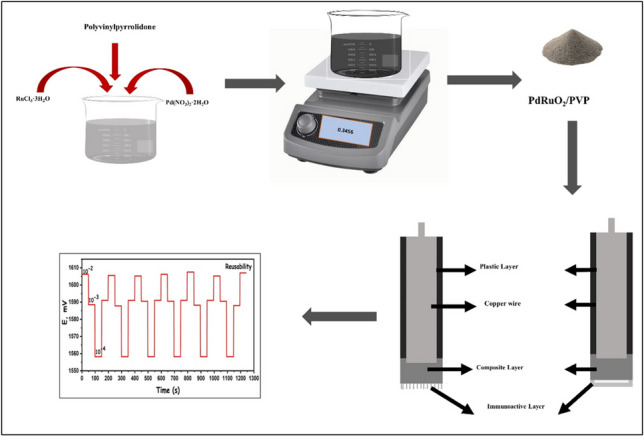

## Introduction

Chromium is used in many industrial activities and is involved in the structural activities of living organisms. Generally, chromium is found in nature in the structure of compounds at Cr(VI) and Cr(III) oxidation levels [1, 2]. Because of its many uses such as wood painting, leather layering, metallurgy, chrome plating, spray painting, and electroplating, chromium is released into the environment as wastewater because of industrial activities [3, 4]. Being a heavy metal, the release of chromium into the environment causes metal pollution. Also, as an essential metal ion for living organisms, Cr(III) plays a vital role in the metabolic reaction of structural molecules of carbohydrates, fat, and protein. The absence of Cr(III) in the human body causes some illnesses such as diabetes, sugar metabolic disorder, cataracts, cardiovascular disease, and blindness [1]. Also, intake excessively of Cr(III) results in several disorders such as vomiting, cancer, diarrhea, nausea, and bleeding in the human body. Therefore detection of Cr(III) in different liquid samples is very important [5]. So far, several methods like colorimetric, chemiluminescence, coupled plasma spectroscopy, fluorescence resonance, X-ray fluorescence, atomic absorption spectrometry (AAS), high-performance liquid chromatography (HPLC) have been used for the detection of Cr(III) [6, 7]. Despite the wide usage of the mentioned methods, they have many disadvantages such as laborious sample preparation, cost, and the need for special equipment. Despite that, potentiometric sensors are efficient, environmentally compatible, low-cost, and versatile. Therefore, potentiometric sensors are seen as an alternative to these methods [4, 8–10]. Additionally, compared to the common techniques, chemical sensors have gained considerable importance due to having several advantages such as easy sample preparation, wide analysis range, simple device use, and good selectivity [11, 12]. Therefore, preparing a suitable electrode material is crucial to the sensor’s activity.

One of the problems encountered in electrode development is that the material used clumps in the solvent and binds poorly to the electrode. This weakens the modification in the resulting electrode and limits the application of the electrode. To overcome this problem, it is necessary to use a material with high solubility. In other words, it is necessary to use a building material that can easily disperse in the solvent environment and form a film on the electrode surface. Polyvinylpyrrolidone (PVP) is a polymeric substance that is highly soluble, easily dispersed, film-forming, non-toxic, and strongly adhesive [13].

Accurate determination of the amount of Cr(III) found in many biological systems and industrial samples is essential because of its widespread availability in many sources. Therefore, the development of biosensors that measure selective, fast, and sensitive chromium facilitates the detection of ions like chromium [14, 15]. To date, various sensors have been prepared for different purposes using different materials [16]. In some studies, some electrode materials have been developed for the detection of various drugs in samples [17, 18]. In addition, there are many different fields of study such as nanocomposite materials, wearable device production, thin film development, and antioxidant activity [19–23]. In some studies, a synergistic effect was created by loading materials such as Fe and MIL-100 on the PVP surface. With this effect, materials with high surface area, high electronic conductivity, and high electrocatalytic properties have been obtained [24].

Modifying relatively cheaper materials with precious metals (Pd, Ru, Pt, Re) can improve the response and selectivity of the resulting sensor to the target substance. Ruthenium (Ru)-based sensors made with PVP and sensitive to Hg^+2^ and Ag^+1^ ions in an EDTA environment were developed by Zhao et al. [25]. In these studies, Ru-supported PVP nanoparticles were obtained homogeneously and the simultaneous detection of these two ions was successfully achieved. In a study, Pd-doped CeO_2_ nanoparticles were prepared and used as gas sensors in a methanol environment, and different amounts of gas were detected successfully by keeping the Pd ratio at 3% [26]. As mentioned above, one of the biggest problems in synthesizing potentiometry sensor materials is ensuring that the synthesized material is dispersed in a suitable solvent environment and adheres to the electrode surface. Although it is possible to prepare homogeneously distributed materials, it is very difficult to detect the desired target molecule or ion accurately. To overcome existing problems, it is necessary to choose a suitable material that can detect the desired target ion. Generally, advanced analytical methods such as TEM, XRD, and SEM are used to reveal the chemical and morphological properties of the materials used in the structure of the electrode. In this study, PdRuO_2_/PVP nanomaterials were synthesized using the reduction/impregnation method, their morphological and structural properties were revealed with some advanced analytical methods and were used effectively in the detection of Cr(III). The nanocluster of Pd and Ru metals on the supporting material (PVP) was investigated by TEM analysis. The crystalline structure was revealed by XRD analysis and surface/content properties were investigated by SEM–EDS. Here, for the first time, PdRuO_2_/PVP nanomaterials were synthesized using a simple method and used as potentiometric sensors. For the first time, PdRuO_2_/PVP nanomaterial was applied successfully for the detection of Cr(III) in sensor studies.

## Material and methods

### Chemicals

RuCl_3_·3H_2_O, Pd(NO_3_)_2_·2H_2_O, polyvinylpyrrolidone (PVP), sodium borohydride, Cr(NO_3_)_3_·9H_2_O, ortho-nitrophenol octyl ether (o-NPOE), dibutyl phthalate (DBP), bis(2-ethylhexyl) adipate (DEHA), bis(2-ethylhexyl)sebacate (BEHS), high relative molecular weight PVC, ethylenediaminetetraacetic acid (EDTA), sodium tetraphenylborate (NaTPB), tetrahydrofuran (THF), graphite, spatula -forceps, epoxy, sodium hydroxide (NaOH), centrifuge—transparent—skirted—screw cap -tube 1.5.

### Instruments

TEM analyses were performed with a TEM Hitachi HT7800 device capable of scanning the elemental range of Boron-Uranium (5B-92 U) with a voltage of 120 kV. XRD analyses were carried out with a Panalytical Zetium brand device. SEM–EDS analyses were performed with a Hitachi Regulus 8230 model device. All potentiometric measurements of Cr^3+^ were performed with a computer-controlled Medisen brand potentiometric sensor device. A glass Ag/AgCl electrode containing saturated 3 M KCl was used as the reference electrode. The elemental mapping analyses were performed by Hitachi Regulus 8230 operating in a range of 0.5–30 kV, a resolution of 1 kV in 0.9. The XPS analyses were implemented by X-ray photoelectron Spectrometer (XPS) device operating at 2 min 35 total acquisition time, Al K Alpha source gun type, 300 µm spot size.

### PdRuO_2_ supported PVP nanoparticles synthesis (PdRuO_2_/PVP)

The synthesis of PdRuO_2_/PVP nanocomposite was carried out using the reduction impregnation method as stated in the literature [27], summarized as follows. Pd(NO_3_)_2_ hydrate and RuCl_3_ hydrate were used as precursors for the synthesis. 2% metal material of ruthenium and palladium and 200 mg PVP as a support material in the synthesis. Firstly, a solution containing PVP (200 mg) and 2% Pb-Ru was prepared in 5-mL distilled water. Then, the resulting mixture was stirred at room conditions and 600 rpm for 2 h. Then, sodium boron hydride, 15 times the mole of metal used, was added to the resulting mixture. After reduction is achieved, the solid part is filtered and washed with plenty of pure water allowed to dry at 120 °C, and then stored for use as electrode material.

### Preparation of PdRuO_2_/PVP nanocomposites-based electrode

Firstly, PdRuO_2_/PVP was prepared in a membrane form to apply to the surface of the electrode. For this, a 100-mg mixture containing ionophores (PdRuO_2_/PVP), graphite, PVC, epoxy, and hardener components was prepared to be used in the composition of the electrodes. Approximately 3 mL of THF was added to the composite components prepared in different proportions and mixed until a homogeneous mixture was obtained. The composites in THF were vortexed to homogenize and mixed for approximately 1–2 min. After ensuring the appropriate viscosity, PdRuO_2_/PVP membrane sensors were prepared by dipping the tip parts of the copper wires prepared the night before into the composites. The prepared biosensors were dried in a dark environment for approximately 12–15 h and kept in a dark environment for taking measurements. The prepared membranes were applied to the surface of the electrodes and left for 12 h to dry. The dried electrodes were kept in 10^−3^ M Cr^3+^ solution for 1 h to condition. Solutions that were not used in subsequent studies were kept in room conditions and a dark environment. Table 1 shows the composition electrode used in the detection of Cr^3+^ ions. Ionophores with different properties were prepared by taking different amounts of substances. The obtained ionophore and *R*^2^ values are given in Table 1. As can be seen from the obtained values, the PdRuO_2_/PVP-based ionophore we prepared gave the best *R*^2^ value.

**Table 1:** The content of PdRuO_2_/PVP based-electrodes, detection limit values, and *R*^2^ data

	PdRuO_2_/PVP (mg)	PVC (mg)	Graphite (mg)	Epoxy (mg)	Hardener (mg)	NaTpClPB (mg)	Detection limit (mol L^−1^)	*R*^2^
1	5.00	31.00	64.00	**-**	-	1.00	7.01 × 10^−6^	0.8806
2	10.00	31.00	62.00	-	-	1.00	6.71 × 10^−6^	0.8978
3	15.00	31.00	60.00	**-**	-	1.00	7.92 × 10^−6^	0.9225
4	20.00	31.00	-	-	64.00	1.00	8.3 × 10^−8^	0.9990
5	25.00	31.00	-	**-**	62.00	1.00	1.81 × 10^−6^	0.9900

### Potentiometric sensor measurements of Cr^3+^

**Fig. 1:**
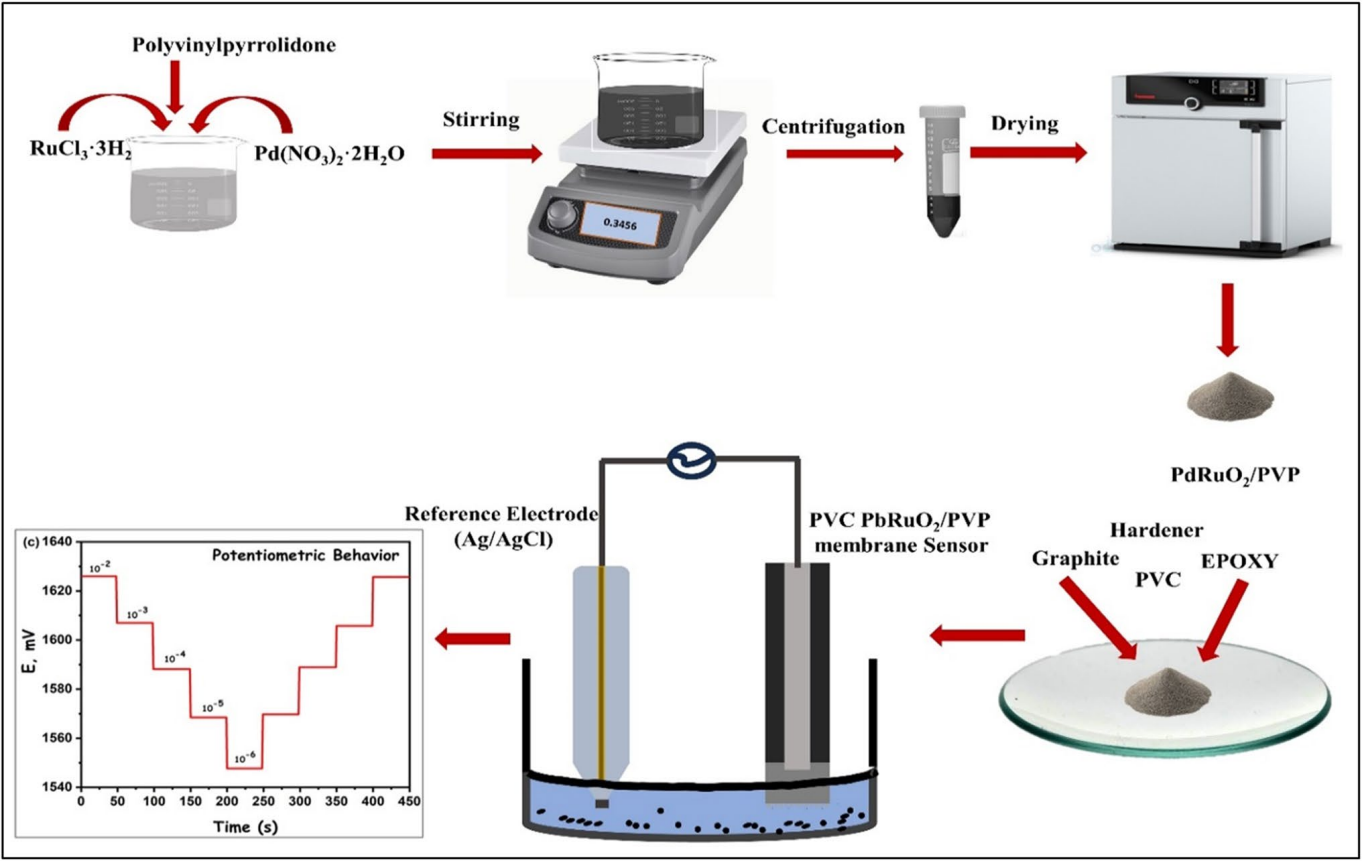
Schematic representation of fabrication of PdRuO_2_/PVP potentiometry sensor

All experiments were carried out at room temperature, 10^−2^ M solution concentration, 1000–1800 Volt potentiometer common parameters. The experimental setup used in the measurements is given in Fig. 1. Measurements were carried out by bringing the reference electrode and working electrodes to the same level in Cr^3+^ solutions of various concentrations (10^−1^–10^−10^ M). In order not to disrupt other solution concentrations in subsequent measurements, the electrode used after the measurements was thoroughly washed with deionized water and dried. Care was taken to ensure that there was no residue from the previous measurement during the measurements.

## Results and discussions

### Characterization studies

#### XRD analyses

**Fig. 2:**
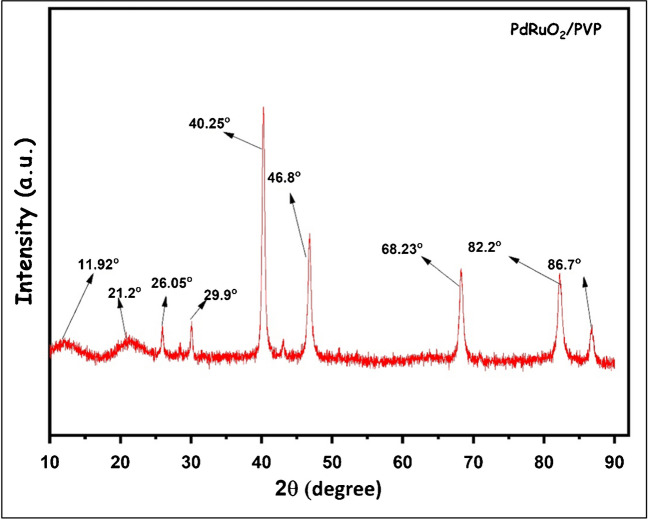
XRD analysis results of PdRuO_2_/PVP potentiometric sensor materials

XRD analysis results of PdRuO_2_/PVP material are given in Fig. 2. As stated in previous studies [28]; the peaks observed at 40.25°, 46.8°, 68.23°, and 82.2° correspond to the (111), (200), (220), and (311) planes for Pd. A peak of 11.92° corresponds to 002 of C present in PVP. Additionally, the peaks seen in the range 21–29° are due to the presence of carbon in the structure of PVP. The mean crystallin particle size was calculated using the Scherrer equation (D = k * λ / β * cos(θ)) and found to be 17.93 nm. The 29.09° seen in the XRD analysis is very close to the specific value of 28.8° for Ru–O [29]. This value indicates the presence of Ru in the structure of PdRuO_2_/PVP. JCPDS card number of XRD analyses of PdRuO_2_/PVP material was found to be 96–154-8201.

**Fig. 3:**
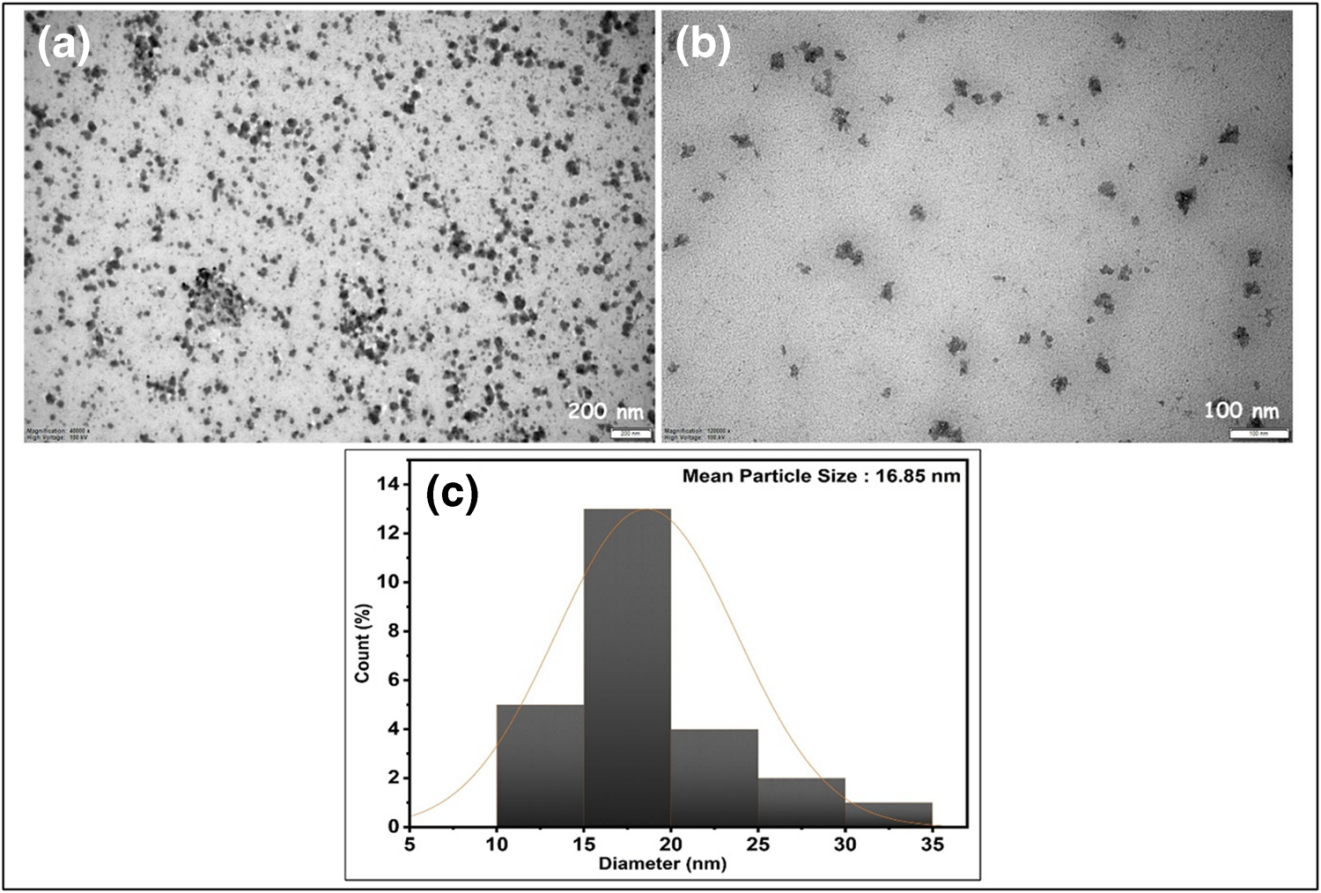
TEM analyses (**a**, **b**) and particle histogram (**c**) of PdRuO_2_/PVP potentiometric sensor materials

Figure 3 shows the TEM analysis results of the PdRuO_2_/PVP material in the 50–200-nm range. It is seen that PdRuO_2_ metal and metal oxides generally have a spherical appearance. The diameters of the spherical atom clusters seen in TEM analyses were measured diagonally. The diameters of the atomic clusters found in TEM analyses were measured from two directions. The obtained values were transferred to the ImageJ program. Then, the average particle size of this value was plotted using the Orgine Lab program. The calculation made using TEM analysis images showed that the mean particle size of PdRuO_2_/PVP material was 16.85 nm. This value obtained is very close to the crystal particle size.

**Fig. 4:**
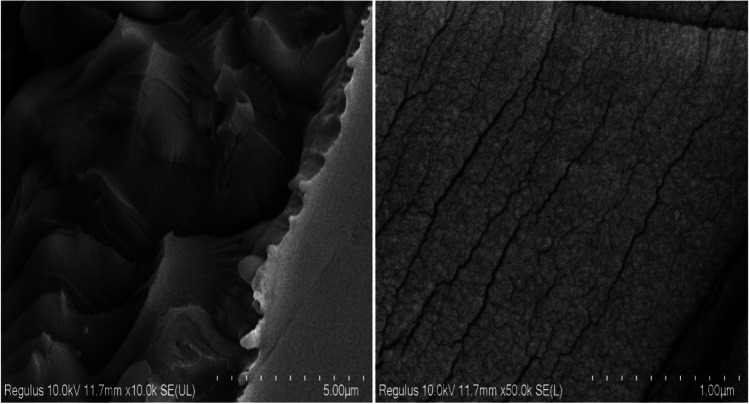
SEM analyses of PdRuO_2_/PVP electrode materials at different scales

SEM analyses were performed to examine the surface structure of the obtained PdRuO_2_/PVP electrode material, and SEM analysis images at different scales are given in Fig. 4. As can be seen from the figures, some porous structures are present in the surface structure. As the surface structure is examined more closely, the pores become clearer in some places. In general, the surface structure has a flat ground appearance.

**Fig. 5:**
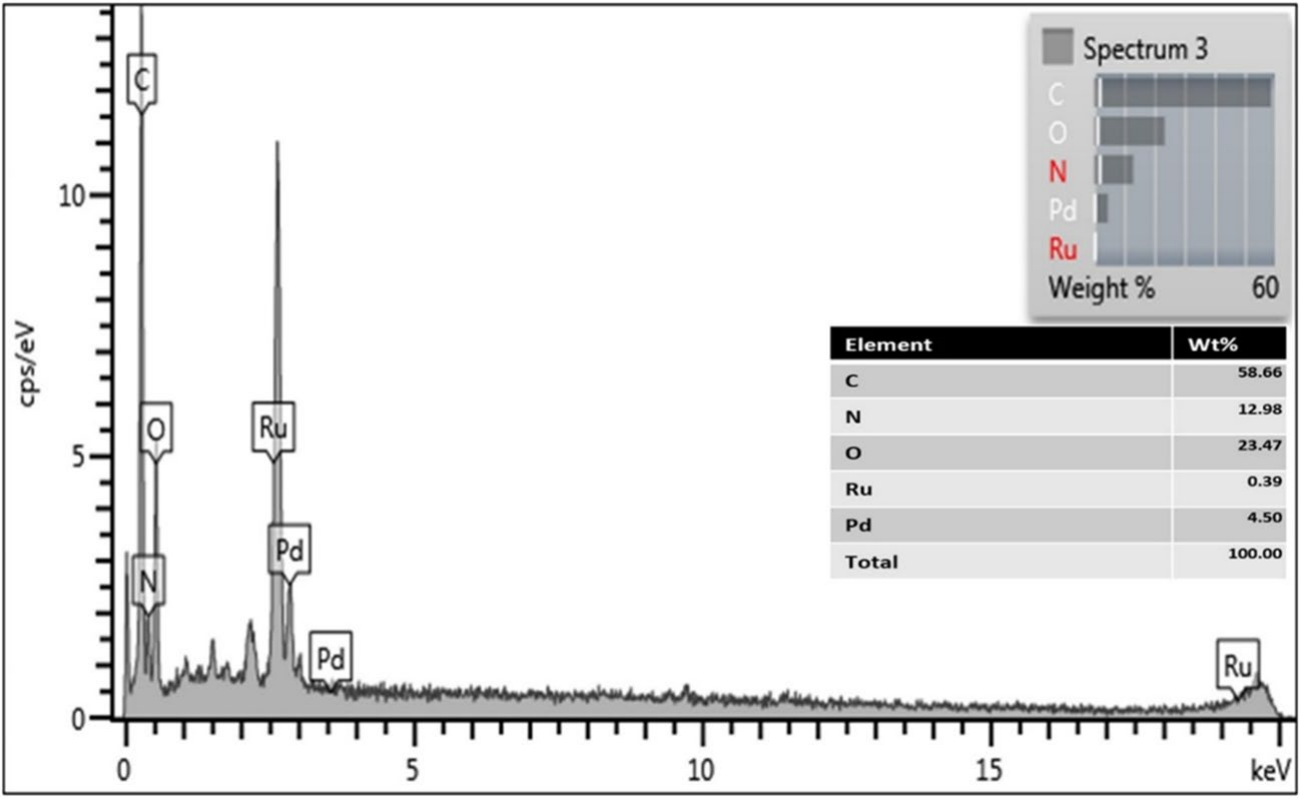
EDS analyses of PdRuO_2_/PVP electrode materials

EDS analyses were performed to examine the chemical composition of the obtained PdRuO_2_/PVP electrode material. As can be seen from the EDS analysis results in Fig. 5, the material obtained contains palladium and ruthenium metals in its structure. It is seen that it contains C and N, which originate from the PVP structure.

**Fig. 6:**
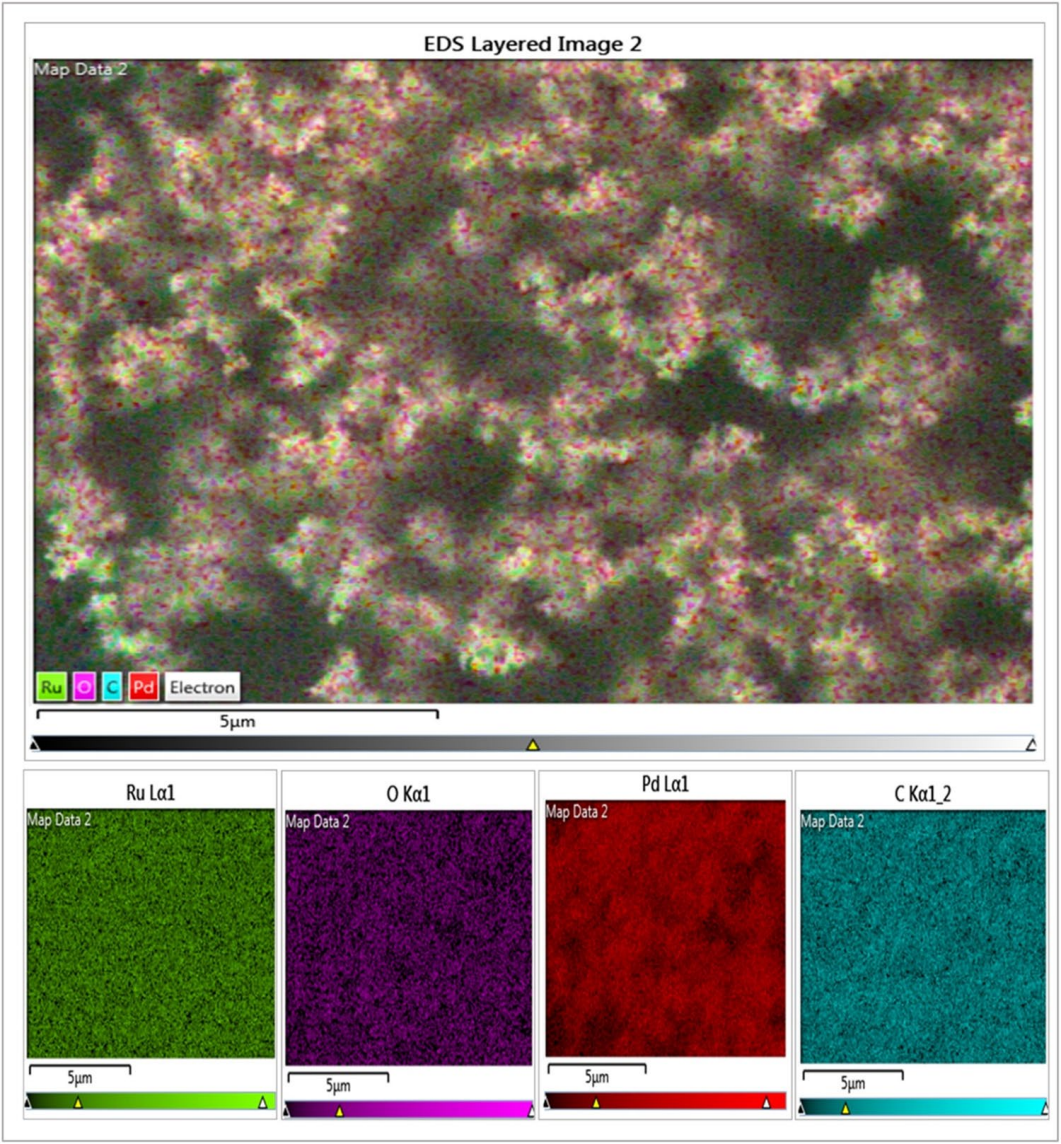
Elemental mapping analyses of PdRuO_2_/PVP electrode materials

Elemental mapping analysis was performed to visualize the elemental composition of the PdRuO2/PVP nanomaterial we synthesized. The results of the elemental mapping analysis are given in Fig. 6. As can be seen, the surface part of the material is composed of Pd, Ru O, and C elements, and it is seen that Pd, Ru, and oxygen elements are evenly distributed on the surface of the nanomaterial.

**Fig. 7:**
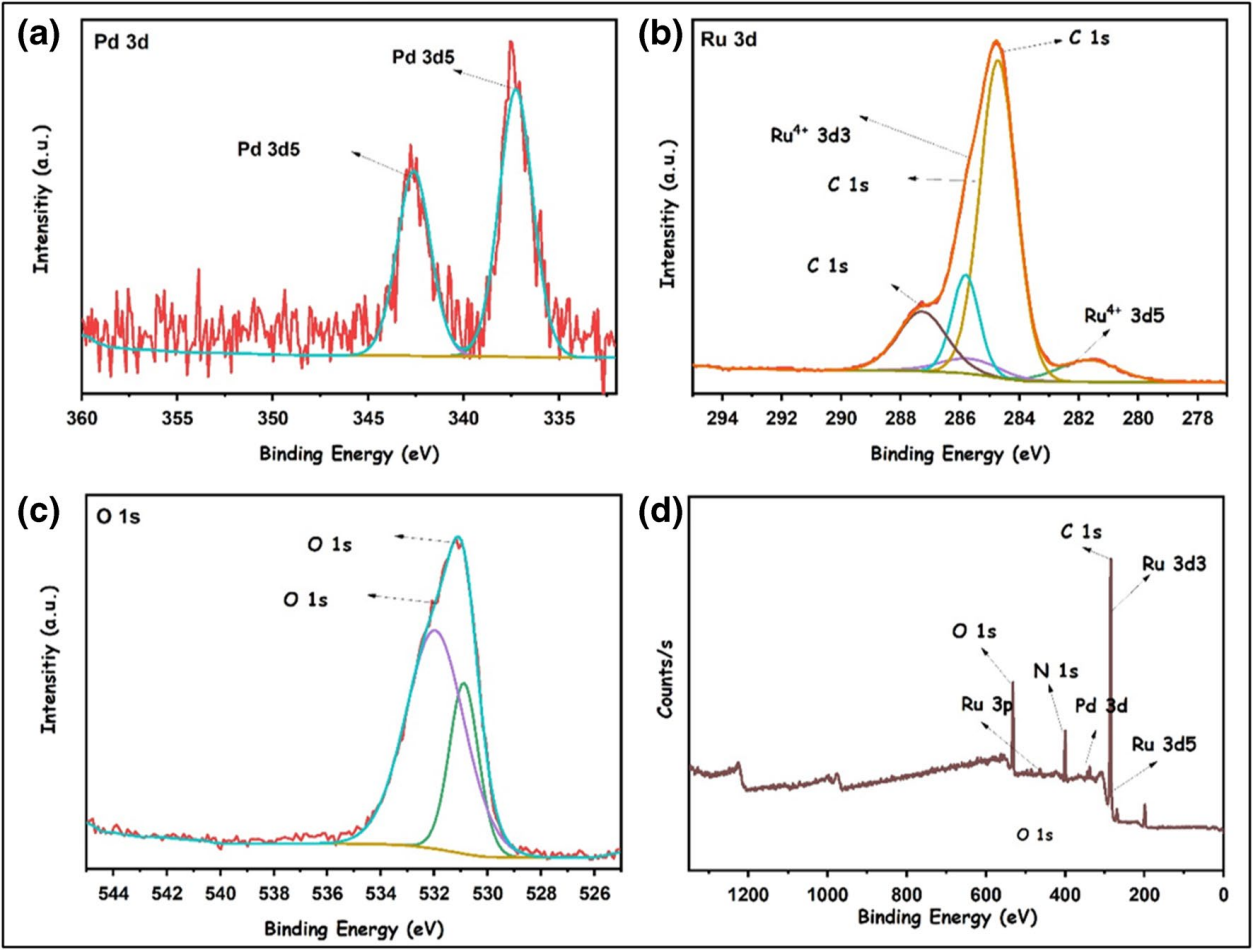
XPS analyses of PdRuO_2_/PVP electrode materials, palladium (**a**), ruthenium (**b**), oxygen (**c**), and a survey (**d**)

XPS analyses were performed to obtain information about the oxidation states of the elements in the PdRuO_2_/PVP material and the chemical environment of these elements. XPS analysis results of PdRuO_2_/PVP material are given in Fig. 7. In the XPS analysis of Ru 3d given in Fig. 7b, the binding energies of 282 and 285.5 eV correspond to 3d5 and 3d3 of Ru^4+^[30]. As can be seen in Fig. 7d, there is no visible impurity due to the element added to the catalyst. O 1 s XPS analyses (Fig. 7c) show that the binding energies of oxygen are at 532 and 531 eV. The presence of RuO_2_ in the material structure increased the power at the O 1 s peaks [31].

The binding energies of 335–336.5 eV, which are characteristic of metallic Pd, are seen in Fig. 7a. The 342 value deviated slightly from 340 eV, which is specific for metallic Pd. Such situations can be observed during XPS analysis because of oxidation or Pd interactions with RuO_2_. Such results have also been observed in other studies [32–34].

## Potentiometric measurements of Cr^3+^ using PdRuO_2_/PVP-based sensor

### Detection limit, linear range, and slope

**Fig. 8:**
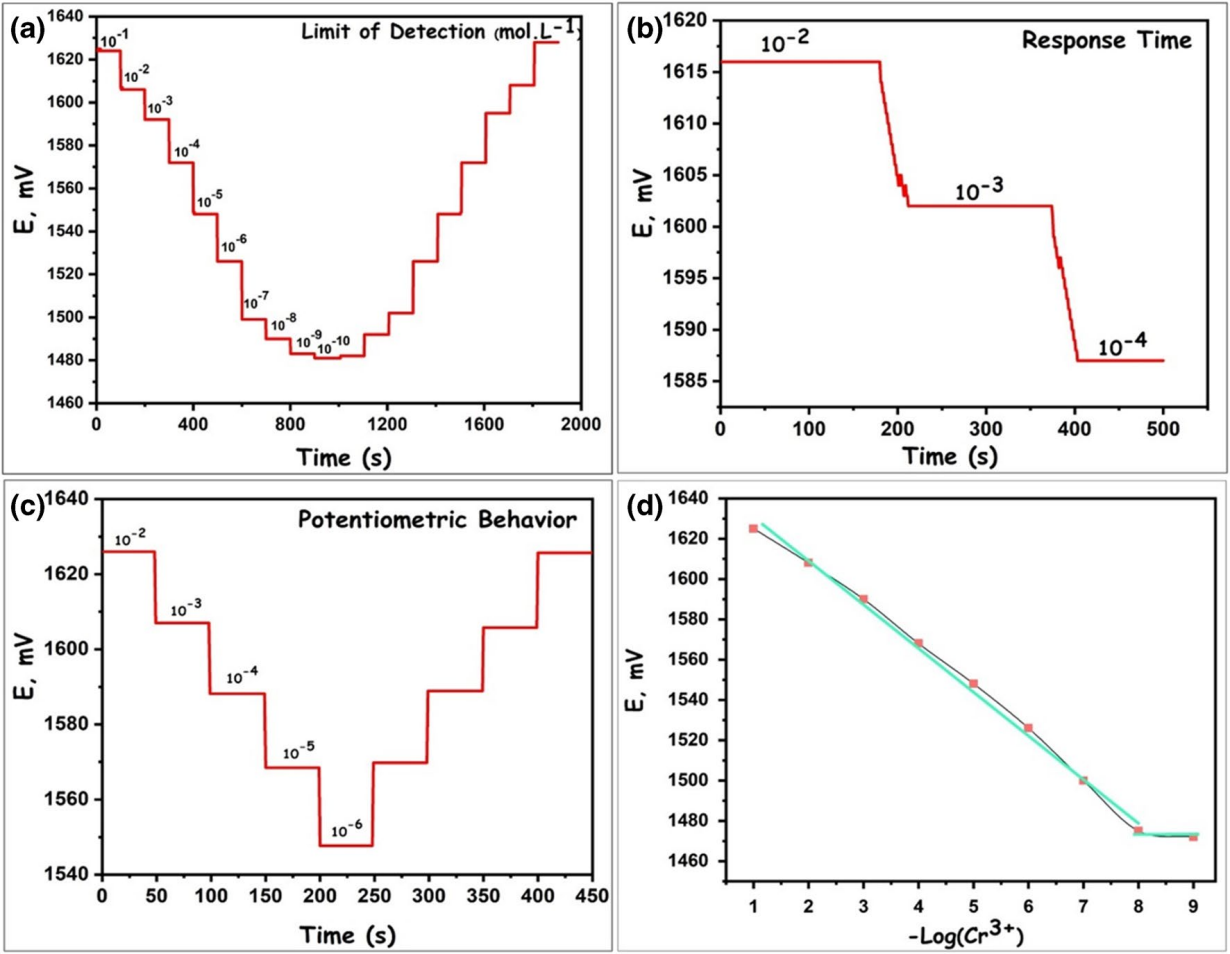
Potentiometric experimental results; limit of detection (**a**), response time (**b**), potentiometric behavior (**c**), and slope of potential

Potentiometric measurements of Cr^3+^ selective PdRuO_2_/PVP electrodes were carried out in 1.10^−6^–1.10^−2^ M Cr^3+^ solutions. The results of the potentiometric measurements obtained and the calibration curves created using these measurements are given in Fig. 8. As a result of the measurements obtained with PdRuO_2_/PVP electrodes in 1.10^−6^–1.10^−2^ M tamoxifen solutions, a linear graph was formed and the slope of this graph was found to be 20.6 ± 0.2 mV/slope Fig. 8d. In addition, the detection limit was expanded by taking more measurements with 1.10^−7^–1.10^−10^ M solutions (Fig. 8a). Thus, the detection limit was obtained more accurately. Using the intersections in the two parts of the extrapolation in the calibration plot given elsewhere, the detection limit was found to be 8.3 ± 0.4. × 10^−8^ M. Figure 8c shows the results of experiments at 10^−2^–10^−6^ M that show the potentiometric behavior. As seen, a linear potentiometric behavior was obtained with the synthesized sensor.

### Response time

The response time was determined to find the time for the constant potential value of the Cr^3+^ ion of the PdRuO_2_/PVP-based sensor to reach equilibrium. To understand this, the response time parameter was measured by dipping the electrode in a 10^−2^ M solution into the next 10^−3^ M solution and then into a 10^−4^ M solution. As stated in the literature, the time when 95% of the potential value reached equilibrium was considered as the response time. Response time experiments of 10^−2^–10^−4^ M Cr^3+^ solutions with PdRuO_2_/PVP sensors are given in Fig. 8b. As can be seen from the experimental results, the obtained electrode reaches equilibrium quickly and the response time was found to be 5 s.

### Selectivity of PdRuO_2_/PVP biosensor

Selectivity in biosensors is found by measuring the response of the electrodes obtained to the target species in the presence of different species in a solution. In the study, the selectivity of PdRuO_2_/PVP electrodes towards Cr^3+^ in 1.0 × 10^−2^–1.0 × 10^−6^ mol.L^−1^ solutions was measured. Using Eq. (1) [35], the selectivity coefficients of PdRuO_2_/PVP electrodes against Cr^3+^ were calculated according to the E_A_ = E_B_ condition [36]. Potential values of measurements conducted with solutions of different species at concentrations of 1.0 × 10^−2^ mol.L^−1^ were found and the obtained potential values are calculated using Eq. (1) given below;

1 $${logK}_{\text{A},\text{B}}^{\text{pot}}=\frac{({E}_{\text{B }}{- E}_{\text{A}}){Z}_{\text{A}}F}{RTln10} +\left(1-\frac{{Z}_{\text{A}}}{{Z}_{\text{B}}}\right)log{a}_{\text{A}}$$

where, E_A_ = activity of chromium ion, E_B_ = activity of interfering ion, Z_A_ = charge of chromium ion, Z_B_ = charge of interfering ion, R = ideal gas constant, T = temperature (K) and F = Faraday constant. The selectivity coefficient of PdRuO_2_/PVP electrodes towards different species is given in Table 2. As can be seen from the results, the selectivity of the PdRuO_2_/PVP electrode towards Cr^3+^ is quite high compared to other ions.

**Table 2:** The selectivity coefficient of PdRuO_2_/PVP electrodes towards different species

Interfering Ions	${Log K}_{Cr+3, j}^{pot}$	${K}_{Cr+3, j}^{pot}$	Interfering ions	${Log K}_{Cr+3, j}^{pot}$	${K}_{Cr+3,j}^{pot}$
Zn^2+^	− 3.405	3.93 × 10^−04^	K^+^	− 3.216	6.07 × 10^−04^
Pb^2+^	− 7.052	8.86 × 10^−08^	Al^+3^	− 4.948	1.13 × 10^−05^
Sr^2+^	− 4.713	1.94 × 10^−05^	Bi^+3^	− 5.257	5.52 × 10^−06^
Cu^2+^	− 2.786	1.63 × 10^−03^	Na^+^	− 3.835	1.46 × 10^−04^
Co^2+^	− 4.644	2.27 × 10^−05^	Mg^+2^	− 7.499	3.16 × 10^−08^
Li^+^	− 2.786	1.63 × 10^−03^	Cd^+2^	− 7.396	4.01 × 10^−08^
Ca^+2^	− 7.809	1.55 × 10^−08^	Ni^+2^	− 7.740	1.82 × 10^−08^
Hg^+2^	− 2.717	1.91 × 10^−03^			

Measurements carried out with PdRuO_2_/PVP potentiometric sensors showed that the Cr^3+^ ion is 103 times more selective than interfering Li^1+^ and Hg^2+^ ions and 108 times more selective than the farthest interfering Ca^2+^ ion. As can be seen from the data in Table 2, the PdRuO_2_/PVP-based sensor showed a potentiometric effect only against Cr. When other results were examined, it was revealed that the PdRuO_2_/PVP nanomaterial, which is a Cr^2+^ sensor, is 108 times more selective towards other ions.

### Reusability and lifespan of PdRuO_2_/PVP electrodes

**Fig. 9:**
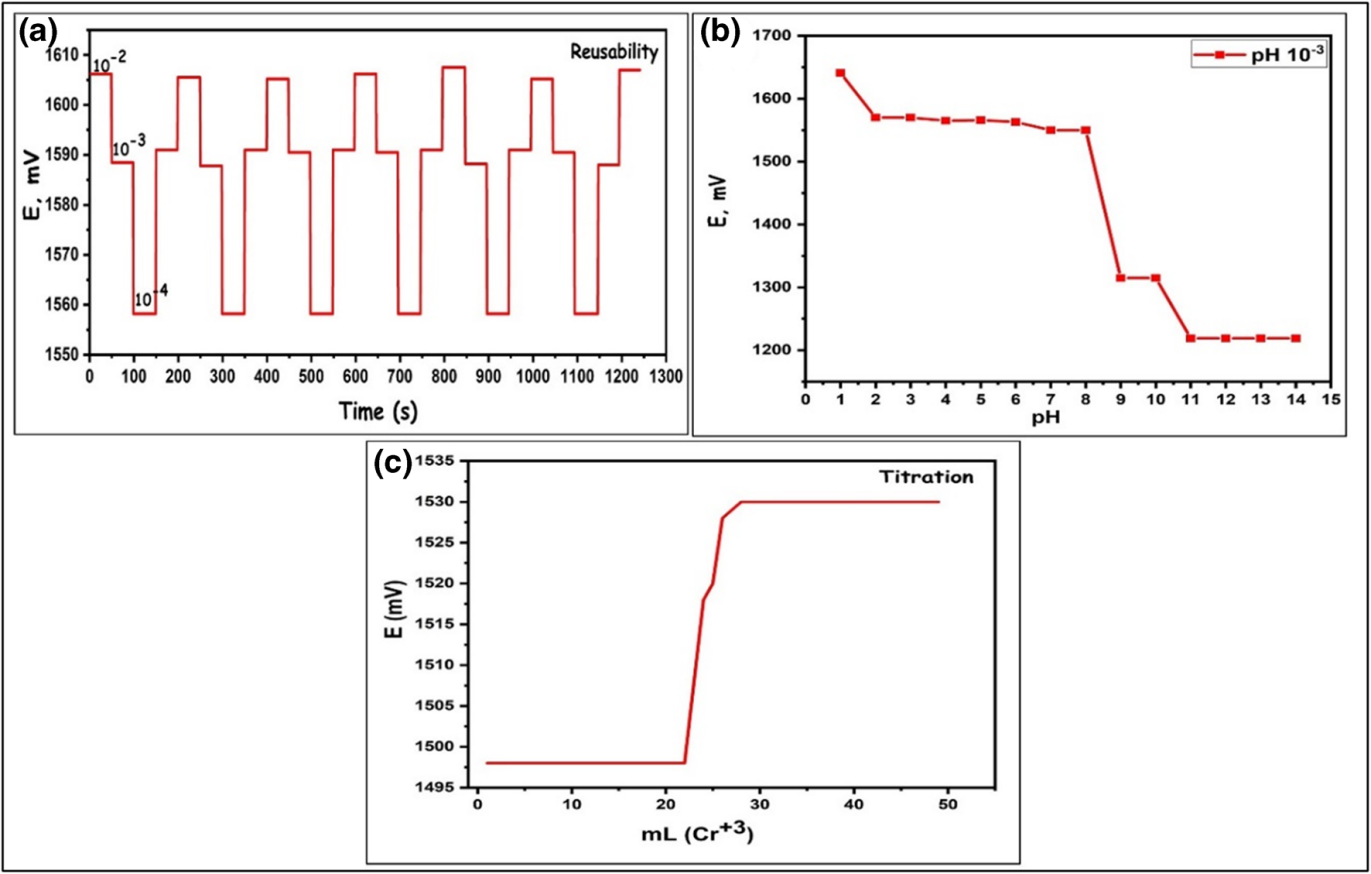
The reusability result of PdRuO_2_/PVP electrodes in 1.0 × 10^−2^ M Cr^3+^ solution (**a**), the responses of PdRuO_2_/PVP -potentiometric electrodes to Cr^3+^ at different pH values (**b**), and the titration experimental results of Cr^3+^ using PdRuO_2_/PVP electrodes

**Fig. 10:**
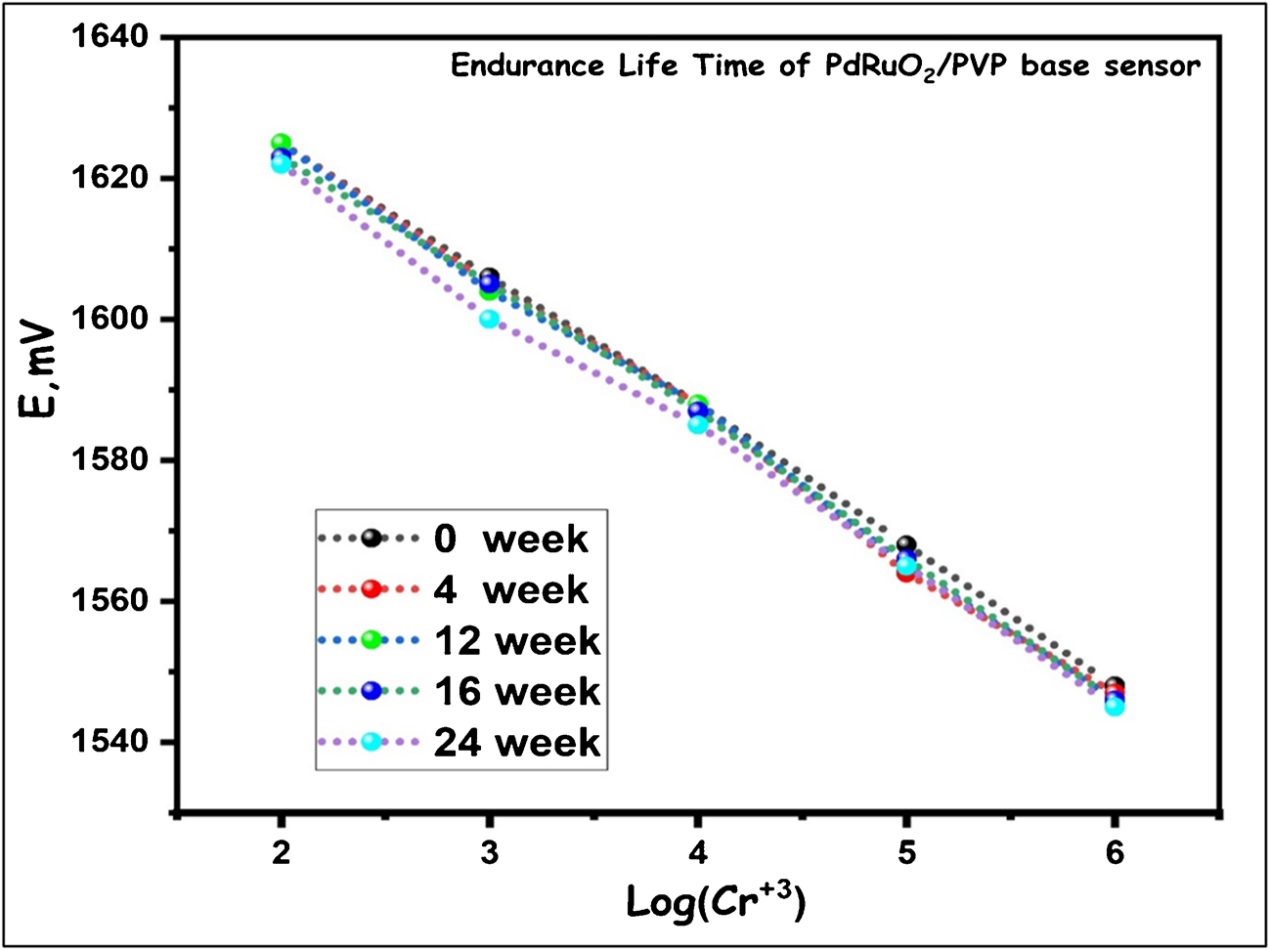
Endurance lifetime of PdRuO_2_/PVP base potentiometric sensor

The experiments of the reusability and lifetime of PdRuO_2_/PVP electrodes were performed in 1.0 × 10^−2^ M, 1.0 × 10^−3^, and 1.0 × 10^−4^ M Cr^3+^ M Cr^3+^ solution. The reusability results of PdRuO_2_/PVP electrodes are given in Fig. 9a. Eight repeated measurements were taken in each solution. The average potential values and standard deviations obtained in 1.0 × 10^−2^ M, 1.0 × 10^−3^ and 1.0 × 10^−4^ M Cr^3+^ M solutions were found to be 1606.2 ± 0.9 mV, 1588.2 ± 1.9 mV, and 558.4 ± 0.8 mV, respectively. As can be seen from the results, the repeatability of PdRuO_2_/PVP electrodes is quite good. These results show that there is almost no change in the measurement efficiency of the PdRuO_2_/PVP electrode after 8 measurements. The lifetime of PdRuO_2_/PVP was obtained in standard prepared solutions by daily calibration and by calculating the slopes in linear ranges. The obtained values indicate that the lifespan of the prepared electrodes exceeds 1 year. It was observed that there was almost no change (less than 2%) in the calibration curves obtained for PdRuO_2_/PVP electrodes over 1 year. Figure 10 depicts the test results of the sensor that was kept for 1 year. As can be seen from the results, it was determined that the obtained sensor had a very stable structure.

1.0 × 10^–2^ mol.L^−1^ Cr^3+^ solutions were used to detect the response of PdRuO_2_/PVP potentiometric electrodes to Cr^3+^ at different pH values. pH ranges were adjusted to 1.0–14.0 pH using NaOH and HCl. The findings are given in Fig. 9b and seen in the results of experiments conducted with different solutions at various pHs the potentiometric electrodes give constant pH values in the range of 2.0–8.0.

### Potentiometric titration experiments using PdRuO_2_/PVP electrodes

PdRuO_2_/PVP potentiometric biosensors exhibited selectivity behavior against Cr^3+^ ions in potentiometric titration experiments having 1.0 × 10^−2^ mol L^−1^ Cr^3+^, 1.0 × 10^−2^ mol.L^−1^ EDTA solutions at room temperatures. In real samples, PdRuO_2_/PVP electrodes along with Cr^3+^ ions were used as indicators. The end point of titration experiments was detected by observation of the decreasing Cr^3+^ concentration Fig. 9c. In the titration experiments of Cr^3+^ with the PdRuO_2_/PVP potentiometric sensor, the sensor exhibited an end value of 25.6 mL of Cr^3+^ concentration. A sharp turning point and a standard sigmoid shape were obtained in the resulting titration curves. This shows that the titration results obtained can be used in the determination of Cr^3+^.

### Effectiveness of PdRuO_2_/PVP electrodes

To detect the effectiveness of our developed PdRuO_2_/PVP electrodes, some results of electrodes developed in the literature for the detection of Cr^3+^ ions were investigated as seen in Table 3. Table 3 compares the analytical response characteristics of the chromium(III) ion-selective sensor we designed with those of many previously reported Cr^3+^ potentiometric sensors. The comparison studies were conducted by taking into account some values of limit detection, pH, concentration, and response time of electrodes. As seen, compared to the sensors in the literature, the developed PdRuO_2_/PVP potentiometric biosensors have some superior features. While the response time of previously developed sensors to Cr^3+^ was between 10 and 20 s, the response time of PdRuO_2_/PVP sensors is only 5 s. Compared to previous studies, the developed sensor can detect Cr^3+^ ions at much lower limits and in a wider pH range. Also, the sensor gives very good values in comparisons such as detection limit and concentration range.

**Table 3:** Some experimental results of electrodes developed for the detection of Cr^3+^ in literature

Ionophore	Linear range (mol L^−1^)	Response time (s)	pH	Detection limit (mol L^−1^)	*R*^2^	Slope (mV per decade)	Ref
diethyl 2-phthalimide malonate	1.0 × 10^−7^–1.0 × 10^−2^	~ 5	2.9–6.1	8.6 × 10^−6^	-	− 20.6	[37]
The Schiff base macrocycle (H_3_L)	1.0 × 10^−5^–1.0 × 10^−1^	< 15	2.5–3.5	2.6 × 10^−5^	-	− 20.4	[38]
5-bromosalicylaldehyde thiosemicarbazone	1.0 10^−5^–1.0 × 10^–1^	< 5	2.5–3.5	3.96 × 10^−6^	0.9979	-	[39]
4-amino-3-hydrazino-6-methyl-1,2,4-triazine-5-one	1.0 × 10^−6^–1.0 × 10^−1^	< 10	2.7–6.6	5.8 × 10^−7^	-	− 19.7	[14]
Aurin tricarboxylic acid	7.0 × 10^−6^–1.0 × 10^−1^	< 10	3.5–6.5	7.0 × 10^−6^	-	− 19.0	[40]
Poly-o-toluidine zirconium(IV) iodosulphosalicylate	3.16 × 10–6^−1^ × 10^−1^	10	3–7	1.58 × 10^−6^		− 20.4	[41]
Bis-glyoxal bis(2-hydroxy anil)	3.0 × 10^−6^–1.0 × 10^−2^	< 20	2.7–6.5	6.3 × 10^−7^		− 19.89	[42]
PdRuO_2_/PVP	1.0 × 10^−6^–1.0 × 10^−1^	< 5	2.0–8.0	8.3 × 10^−8^	0.9996	− 19.6	This work

Analytical applications of the obtained PdRuO_2_/PVP electrode, which is selective for Cr^3+^, were implemented with different drinks. For this purpose, a certain amount of Cr^3+^ ion was added to known beverage samples, and potentiometric measurements were performed. The measurement results obtained are given in Table 4. The concentrations of Cr^3+^ were calculated by substituting the obtained potential values into the equation in the linear graph. The concentrations found were compared with the Cr^3+^ concentrations added to beverages. These compared values revealed a high amount of recovery and showed that the PdRuO_2_/PVP electrode obtained was an alternative method to traditional analytical methods used for Cr^3+^ ion detection.

**Table 4:** The potentiometric measurement results were performed with different beverages for the detection of Cr^3+^

Beverage samples	Found (mol.L^−1^)	Added (mol.L^−1^)	%R	%R.S.D
Apricot	1.10 × 10^−03^	1.00 × 10^−3^	91.13	2.14
Apricot	1.02 × 10^−04^	1.00 × 10^−4^	97.91	2.09
Apricot	1.02 × 10^−05^	1.00 × 10^−5^	97.64	1.75
Cherries	1.05 × 10^−03^	1.00 × 10^−3^	95.73	4.23
Cherries	1.07 × 10^−04^	1.00 × 10^−4^	93.45	2.08
Cherries	1.07 × 10^−05^	1.00 × 10^−5^	93.17	2.19
Water	1.04 × 10^−03^	1.00 × 10^−4^	95.96	2.14
Water	1.05 × 10^−04^	1.00 × 10^−5^	95.40	3.72

The statistical analysis results of the data of measurements of Cr(III) detection with PdRuO_2_/PVP-based sensor are given in Table 5. Six measurements were performed with solutions having 0.1, 0.01, and 0.0001 M concentrations, and their results were added to the table, and their average and standard deviation were calculated using Excell packet program. The standard deviation value found in statistical calculations is quite low. This value shows that the electrode is quite stable and these results are compatible with the results obtained in repeated measurements.

**Table 5:** The statistical analysis of the data of measurements of Cr(III) detection with PdRuO_2_/PVP-based sensor

Con.(M)	1st Meas	2nd Meas	3rd Meas	4th Meas	5th Meas	6th Meas	Average	Standard deviation
0.01	1606.2	1605.6	1605.2	1606.2	1606.9	1605.3	1605.9	0.55032458
0.001	1588.5	1587.8	1589.2	1590.2	1589.2	1588.9	1588.966667	0.67753053
0.0001	1558.2	1558.2	1558.3	1558.4	1558.4	1558.7	1558.366667	0.15735915

## Conclusion

In this study, for the first time, PdRuO_2_/PVP electrode material was successfully synthesized, its chemical structure was elucidated using various advanced analytical methods and was successfully used as a potentiometric sensor in the detection of Cr^3+^ ion. The developed PdRuO_2_/PVP potentiometric sensor was found to have outstanding selectivity towards Cr^3+^ ions among a wide range of interfering ions. PdRuO_2_/PVP electrode was simply and efficiently used as a suitable sensing element (ionophore) as a selective potentiometric sensor. The sensor is highly sensitive with a detection limit of 8.6 × 10^−8^ mol.L^−1^ for the rapid detection of chromium(III) ions in the range of 1 × 10^−6^–1.0 × 10^−1^ M in aqueous samples. The easy structure and selective and fast response of the developed detection device enable PdRuO_2_/PVP to be used in the routine analysis of various real aqueous matrices. To the best of our knowledge, the developed PdRuO_2_/PVP sensor is one of the most selective and sensitive potentiometric sensors reported so far for the rapid and accurate detection of ultra-trace amounts of Cr^3+^ in industrial, environmental, and biological samples. The features such as linear range, detection limit, selectivity, operating pH range, and lifetime clearly showed that the proposed PdRuO_2_/PVP-based sensor can be classified as the best Cr^3+^ sensor among the reported sensors, especially in terms of high selectivity and least interfering ion. Also, having strong repeatability and 1 year lifetime revealed that PdRuO_2_/PVP electrode material is extremely suitable for the detection of Cr^3+^ ions in liquid samples.

## Data Availability

The datasets generated during and/or analyzed during the current study are available from the corresponding authors on reasonable request.

## References

[1] Mahato P, Saha S, Suresh E et al (2012) Ratiometric detection of Cr 3+ and Hg 2+ by a naphthalimide-rhodamine based fluorescent probe. Inorg Chem 51:1769–1777. 10.1021/IC202073Q 22235801 10.1021/IC202073Q

[2] Zhao M, Ma L, Zhang M et al (2013) Glutamine-containing “turn-on” fluorescence sensor for the highly sensitive and selective detection of chromium (III) ion in water. Spectrochim Acta Part A Mol Biomol Spectrosc 116:460–465. 10.1016/J.SAA.2013.07.069 10.1016/J.SAA.2013.07.069 23973594

[3] Bohrn U, Mucha A, Werner CF et al (2013) A critical comparison of cell-based sensor systems for the detection of Cr(VI) in aquatic environment. Sensors Actuators B Chem 182:58–65. 10.1016/J.SNB.2013.02.105 10.1016/J.SNB.2013.02.105

[4] Salimi A, Pourbahram B, Mansouri-Majd S, Hallaj R (2015) Manganese oxide nanoflakes/multi-walled carbon nanotubes/chitosan nanocomposite modified glassy carbon electrode as a novel electrochemical sensor for chromium (III) detection. Electrochim Acta 156:207–215. 10.1016/J.ELECTACTA.2014.12.146 10.1016/J.ELECTACTA.2014.12.146

[5] Kumar P, Sharma HK (2013) Development of all solid state chromium(III) selective sensor by using newly synthesized triazole derivative as an ionophore in PVC matrix. Electrochim Acta 87:925–929. 10.1016/J.ELECTACTA.2012.09.027 10.1016/J.ELECTACTA.2012.09.027

[6] Kimbrough DE, Cohen Y, Winer AM et al (1999) A critical assessment of chromium in the environment. Crit Rev Environ Sci Technol 29:1–46. 10.1080/10643389991259164 10.1080/10643389991259164

[7] Singh A, Walker KJ, Sijwali PS et al (2007) A chimeric cysteine protease of Plasmodium berghei engineered to resemble the Plasmodium falciparum protease falcipain-2. Protein Eng Des Sel 20:171–177. 10.1093/PROTEIN/GZM009 17430972 10.1093/PROTEIN/GZM009

[8] Zhang N, Suleiman JS, He M, Hu B (2008) Chromium(III)-imprinted silica gel for speciation analysis of chromium in environmental water samples with ICP-MS detection. Talanta 75:536–543. 10.1016/J.TALANTA.2007.11.059 18371918 10.1016/J.TALANTA.2007.11.059

[9] Karak D, Banerjee A, Sahana A et al (2011) 9-Acridone-4-carboxylic acid as an efficient Cr(III) fluorescent sensor: trace level detection, estimation and speciation studies. J Hazard Mater 188:274–280. 10.1016/J.JHAZMAT.2011.01.110 21345582 10.1016/J.JHAZMAT.2011.01.110

[10] de Paula CER, Caldas LFS, Brum DM, Cassella RJ (2013) Development of a focused ultrasound-assisted extraction method for the determination of trace concentrations of Cr and Mn in pharmaceutical formulations by ETAAS. J Pharm Biomed Anal 74:284–290. 10.1016/J.JPBA.2012.11.013 23245262 10.1016/J.JPBA.2012.11.013

[11] Chen XV, Bühlmann P (2022) Ion-selective potentiometric sensors with silicone sensing membranes: a review. Curr Opin Electrochem 32:100896. 10.1016/J.COELEC.2021.100896 10.1016/J.COELEC.2021.100896

[12] Lisak G (2021) Reliable environmental trace heavy metal analysis with potentiometric ion sensors - reality or a distant dream. Environ Pollut 289:117882. 10.1016/J.ENVPOL.2021.117882 34364114 10.1016/J.ENVPOL.2021.117882

[13] Yu T, Wang Y, Yuan K et al (2022) Electrochemical immunosensor with PVP/WS2 nanosheets electrode for fibroblast growth factor 21 detection based on metal organic framework nanozyme. Sensors Actuators B Chem 367:132056. 10.1016/J.SNB.2022.132056 10.1016/J.SNB.2022.132056

[14] Zamani HA, Rajabzadeh G, Ganjali MR (2006) Highly selective and sensitive chromium(III) membrane sensors based on 4-amino-3-hydrazino-6-methyl-1,2,4-triazin-5-one as a new neutral ionophore. Sensors Actuators B Chem 119:41–46. 10.1016/J.SNB.2005.11.048 10.1016/J.SNB.2005.11.048

[15] Reza GM, Ali ZH, Parviz N, Mehdi A, Morteza R, Aceedy M (2005) Zn2+PVC-based membrane sensor based on 3-[(2-Furylmethylene)amino]-2-thioxo-1,3-thiazolidin-4-one. Bull Korean Chem Soc 26:579–584 10.5012/bkcs.2005.26.4.579

[16] Lu Y, Wang Z, Mu X et al (2022) The electrochemical sensor based on Cu/Co binuclear MOFs and PVP cross-linked derivative materials for the sensitive detection of luteolin and rutin. Microchem J 175:107131. 10.1016/J.MICROC.2021.107131 10.1016/J.MICROC.2021.107131

[17] Ansari S, Ansari MS, Satsangee SP, Jain R (2019) WO3 decorated graphene nanocomposite based electrochemical sensor: a prospect for the detection of anti-anginal drug. Anal Chim Acta 1046:99–109. 10.1016/J.ACA.2018.09.028 30482307 10.1016/J.ACA.2018.09.028

[18] Ansari S, Ansari MS, Devnani H et al (2018) CeO2/g-C3N4 nanocomposite: a perspective for electrochemical sensing of anti-depressant drug. Sensors Actuators B Chem 273:1226–1236. 10.1016/J.SNB.2018.06.036 10.1016/J.SNB.2018.06.036

[19] Sharma C, Ansari S, Ansari MS et al (2020) Single-step green route synthesis of Au/Ag bimetallic nanoparticles using clove buds extract: enhancement in antioxidant bio-efficacy and catalytic activity. Mater Sci Eng C 116:111153. 10.1016/J.MSEC.2020.111153 10.1016/J.MSEC.2020.111153 32806256

[20] Ansari MS, Othman MHD, Ansari MO et al (2020) Room temperature growth of half-metallic Fe3O4 thin films on polycarbonate by reactive sputtering: heterostructures for flexible spintronics. J Alloys Compd 816:152532. 10.1016/J.JALLCOM.2019.152532 10.1016/J.JALLCOM.2019.152532

[21] Ansari MS, Othman MHD, Ansari MO et al (2020) Reactively sputtered half-metallic Fe3O4 thin films at room temperature on polymethyl methacrylate: a perspective for flexible spintronics. Ceram Int 46:19302–19310. 10.1016/J.CERAMINT.2020.04.270 10.1016/J.CERAMINT.2020.04.270

[22] Ansari MS, Othman MHD, Ansari MO et al (2020) Magnetite thin films grown on different flexible polymer substrates at room temperature: role of antiphase boundaries in electrical and magnetic properties. J Alloys Compd 846:156368. 10.1016/J.JALLCOM.2020.156368 10.1016/J.JALLCOM.2020.156368

[23] Ansari MS, Othman MHD, Ansari MO et al (2021) Large spin-dependent tunneling magnetoresistance in Fe3O4/PET heterostructures developed at room temperature: a promising candidate for flexible and wearable spintronics. Mater Sci Eng B 265:115033. 10.1016/J.MSEB.2020.115033 10.1016/J.MSEB.2020.115033

[24] Liu X, Cui G, Dong L et al (2022) Synchronous electrochemical detection of dopamine and uric acid by a PMo12@MIL-100(Fe)@PVP nanocomposite. Anal Biochem 648:114670. 10.1016/J.AB.2022.114670 35367219 10.1016/J.AB.2022.114670

[25] Zhao Y, Yang X, Cui L et al (2018) PVP-capped Pt NPs-depended catalytic nanoprobe for the simultaneous detection of Hg2+ and Ag+. Dye Pigment 150:21–26. 10.1016/J.DYEPIG.2017.11.007 10.1016/J.DYEPIG.2017.11.007

[26] Hu Q, Huang B, Li Y et al (2020) Methanol gas detection of electrospun CeO2 nanofibers by regulating Ce3+/ Ce4+ mole ratio via Pd doping. Sensors Actuators B Chem 307:127638. 10.1016/J.SNB.2019.127638 10.1016/J.SNB.2019.127638

[27] Bayat R, Bingül Reçber Z, Bekmezci M et al (2022) Synthesis and application of AuNi@AC nano adsorbents for the removal of Maxilon Blue 5G azo dye from aquatic mediums. Food Chem Toxicol 167:113303. 10.1016/J.FCT.2022.113303 35850400 10.1016/J.FCT.2022.113303

[28] Zhang Y, Li J, Wang C et al (2023) Activable Ru-PdRu nanosheets with heterogeneous interface for high-efficiency alcohol oxidation reaction. J Colloid Interface Sci 647:519–527. 10.1016/J.JCIS.2023.05.095 37230830 10.1016/J.JCIS.2023.05.095

[29] Rajagopal N, Uppara HP, Dasari H et al (2023) Insights of MOF-derived bimetallic PVP/Ce-Ru nanocomposites for diesel soot oxidation. Inorg Chem Commun 155:111126. 10.1016/J.INOCHE.2023.111126 10.1016/J.INOCHE.2023.111126

[30] Shi X, Du M, Jing H et al (2023) Bold innovation of noble metal support system: Ru-RuO2/MXene@CC for efficient hydrogen evolution reaction in water electrolysis. Colloids Surfaces A Physicochem Eng Asp 679:132638. 10.1016/J.COLSURFA.2023.132638 10.1016/J.COLSURFA.2023.132638

[31] Lee J, Lee S, Kim Y et al (2024) Atomic layer deposited RuO2 with controlled crystallinity and thickness for oxygen evolution reaction catalysis. Vacuum 220:112843. 10.1016/J.VACUUM.2023.112843 10.1016/J.VACUUM.2023.112843

[32] Karhu H, Kalantar A, Väyrynen IJ et al (2003) XPS analysis of chlorine residues in supported Pt and Pd catalysts with low metal loading. Appl Catal A Gen 247:283–294. 10.1016/S0926-860X(03)00098-X 10.1016/S0926-860X(03)00098-X

[33] Dumbuya K, Denecke R, Steinrück HP (2008) Surface analysis of Pd/ZnO catalysts dispersed on micro-channeled Al-foils by XPS. Appl Catal A Gen 348:209–213. 10.1016/J.APCATA.2008.06.037 10.1016/J.APCATA.2008.06.037

[34] Gözeten I, Calimli MH, Nas MS et al (2024) Palladium-loaded on calcined ulexite (Pd(0)@CU) nanoparticles for the catalytic hexavalent chromium reduction. Int J Environ Sci Technol 21:1745–1766. 10.1007/S13762-023-05263-3/TABLES/1 10.1007/S13762-023-05263-3/TABLES/1

[35] Lindner E, Umezawa Y (2008) Performance evaluation criteria for preparation and measurement of macro- and microfabricated ion-selective electrodes (IUPAC Technical Report). Pure Appl Chem 80:85–104. 10.1351/PAC200880010085/MACHINEREADABLECITATION/BIBTEX 10.1351/PAC200880010085/MACHINEREADABLECITATION/BIBTEX

[36] Buck RP, Lindner E (1994) Recomendations for nomenclature of ion-selective electrodes (IUPAC recommendations 1994). Pure Appl Chem 66:2527. 10.1351/pac199466122527 10.1351/pac199466122527

[37] Zamani HA, Sahebnasagh S (2013) Potentiometric detection of Cr 3+ ions in solution by a chromium(III) electrochemical sensor based on diethyl 2-phthalimidomalonate doped in polymeric membrane. Int J Electrochem Sci 8:3708–3720 10.1016/S1452-3981(23)14425-7

[38] Fekri MH, Khanmohammadi H, Darvishpour M (2011) An electrochemical Cr(III)-selective sensor-based on a newly synthesized ligand and optimization of electrode with a nano particle. Int J Electrochem Sci 6:1679–1685 10.1016/S1452-3981(23)15102-9

[39] Isildak Ö, Özbek O, Yildiz I, Senocak A (2023) Selective determination of Cr(III) ions with novel potentiometric electrodes. J Chinese Chem Soc 70:1814–1820. 10.1002/JCCS.202300165 10.1002/JCCS.202300165

[40] Sharma RK, Goel A (2005) Development of a Cr(III)-specific potentiometric sensor using Aurin tricarboxylic acid modified silica. Anal Chim Acta 534:137–142. 10.1016/J.ACA.2004.11.026 10.1016/J.ACA.2004.11.026

[41] Lutfullah RM, Khan F, Wahab R (2014) Poly o -toluidine zirconium(IV) iodosulfosalicylate-based ion-selective membrane electrode for potentiometric determination of Cr(III) ions and its analytical applications. Ind Eng Chem Res 53:14897–14903. 10.1021/ie501788a 10.1021/ie501788a

[42] Gholivand MB, Sharifpour F (2003) Chromium(III) ion selective electrode based on glyoxal bis(2-hydroxyanil). Talanta 60:707–713. 10.1016/S0039-9140(03)00130-9 18969095 10.1016/S0039-9140(03)00130-9

